# Evaluation of Mandibular Bone Microstructure in CT Scans of People with Sickle Cell Disease

**DOI:** 10.1055/s-0043-1771200

**Published:** 2023-09-20

**Authors:** Viviane de Sousa Moreira Almeida, Mariana Quirino Silveira Soares, Patricia Miranda Leite-Ribeiro, Izabel Regina Fischer Rubira-Bullen, Liliane Elze Falcão Lins-Kusterer, Viviane Almeida Sarmento

**Affiliations:** 1 School of Dentistry, Federal University of Bahia, Salvador, Bahia, Brazil; 2 Bauru School of Dentistry, University of São Paulo, Bauru, São Paulo, Brazil; 3 Medical School, Federal University of Bahia, Salvador, Bahia, Brazil

**Keywords:** sickle cell disease, computed tomography, bone changes

## Abstract

**Objective** Sickle cell disease (SCD) is a common inherited disease, and is characterized by a genetic modification that determines the production of a hemoglobin with altered morphology. This anatomical change of hemoglobin leads to vaso-occlusive disorders and premature hemolysis of the cell, causing chronic anemia and bone marrow hyperplasia due to increased hematopoietic demand. As a consequence, several skeletal changes are reported in the skull, spine, ribs, pelvis, femur, and metatarsals. In the craniofacial region, dentofacial deformities are described, especially maxillary protrusion. However, studies evaluating bone microarchitecture are scarce. The aim of this study is to evaluate the mandibular bone microstructure of people with SCD on computed tomography (CT) scans.

**Materials and Methods** Morphometric parameters were analyzed on CT scans of the mandible of people with SCD and people without this disease or any other disease affecting bone metabolism, matched for sex and age.

**Statistical Analysis** The results were compared by Student's *t* -test for paired samples and for an error probability of 5%.

**Results** This study demonstrated that the mandibular bone of people with SCD presents significantly less number, connectivity and thickness of bone trabeculae, as well as having a lower fractal dimension and greater porosity.

**Conclusion** Mandibular bone of people with SCD has lower bone density and more widely spaced trabeculae.

## Introduction

Sickle cell disease (SCD) is a hematologic disease in which a genetic alteration in the formation of hemoglobin (Hb) determines the formation of Hb S, instead of normal Hb A. When in homozygosis (Hb SS), the disease is called sickle cell anemia (SCA), with more evident signs and symptoms. The presence of one Hb S and Hb A determines the sickle cell trait (SCT), when the person does not have the disease, but can transmit it to their descendants; and the presence of Hb S with other Hb alterations (C or D) determines other forms of SCD, generally with milder symptoms. 1 2

Altered Hb is less flexible and adheres to the endothelium, causing vaso-occlusion of the microcirculation, restricting blood flow to multiple organs and systems. 3 The accumulation of these cells is responsible for vaso-occlusion that can cause ischemia, infarcts, and tissue damage. These pathophysiological events determine most of the signs and symptoms of people with toms of people with SCD: painful crises, high susceptibility to infections and bone necrosis, osteopenia, osteoporosis, hemolytic crises, lower limb ulcers, splenic sequestration, priapism, strokes and chronic processes impairment of multiples organs or systems. 4 5 6

Commonly referred to as vaso-occlusive crises, these episodes can occur from infancy to old age, although they increase in frequency throughout childhood, with a peak in the mid-20s. 7

The morphological change of the red blood cells (RBCs) promotes their early hemolysis and thus the bone marrow, especially that of longer bones, increases the production of new RBCs to try to compensate for the anemia and this leads to a bone marrow expansion. This may explain the skeletal changes related to the disease, such as maxillary protrusion, with elongation and rotation of the mandible, determining a class II profile. 8 Additionally, subjective changes in the bone density of the maxillomandibular complex are also reported, such as increased medullary spaces, accentuated bone trabeculation, and denser or more porous areas. 9 10 11

Although the signs of the disease manifest early in the life of the affected person, there are many situations in which the diagnosis of SCD occurs late, contributing to the morbidity of the disease and decreased quality of life of the patient. Thus, clinical or imaging signs are important to help triage suspected patients and indicate tests to confirm the diagnosis.

Despite studies evaluating facial anatomical skeletal changes are found, 8 there are few that have objectively evaluated the bone microarchitecture in people with SCD. The aim of this study is to characterize the microarchitecture of the mandibular bone of people with SCD on computed tomography (CT) examination.

## Materials and Methods

The sample consisted of CT scans of the mandible of people with SCD being treated at University Hospital Professor Edgard Santos (Federal University of Bahia, Salvador, BA, Brazil) from 2014 to 2019, which constituted the test group; and CT scans of the mandible of people without SCD or SCT, matched for sex and age (+2 years), which constituted the control group. CT scans were performed on clinical indication, as part of routine care, with no unnecessary patient exposure to X-rays.

Inclusion criteria were diagnosis of SCD (for the test group), diagnosed by hemoglobin electrophoresis (Hb SS genotype, Hb SC genotype, and β-thalassemia genotype), 18 years or older at the time of CT scanning, CT scans obtained with thin reformatting (no more than 0.5 mm thick), with a bone window, including the entire mandible. Exclusion criteria were presence of bone lesions, fractures, deformities or previous surgery in the mandible, or diseases that affected bone metabolism, or metallic artifacts that hindered the correct evaluation of the images.

The selected scans were archived in electronic media in DICOM (Digital Imaging and Communications in Medicine) format and opened in the software RadiAnt DICOM Viewer (v. 5.01) for exportation of the sequence of bone window sections comprising the area of interest of this study.

Then this sequence was opened in the MeVisLab software, in which the multiple files were grouped into a single file, which was then converted into figure files with extension bmp, in grayscale with eight bits by DicomToCTAn Converter (Medical Imaging Researcher Center, Leuven, Belgium).

Then, in the DataViewer software, the images were aligned and cropped, selecting the region of interest that were located on the body of both sides of each mandible, and saved again. After this step, the images were opened in CTAnalyser software (v. 1.11.10.0, Skyscan, Konitch, Belgium). The volume of interest (VOI) of each exam was standardized, extending from 1 mm before the last image of the mental foramen to 1 mm after this landmark. After establishing the VOI, the images from each side of the mandible were binarized by applying an automatic threshold (the bony structures were shown in red and the medullary spaces in green). Filters provided by the CTAnalyser software itself (v. 1.11.10.0, Skyscan, Konitch, Belgium) were applied for better visualization of the bone trabeculae, and then the selection of the area to be analyzed was performed.

The following parameters of the bone microarchitecture were automatically calculated by the CTAnalyser software: thickness of the bone trabeculae (Tb.Th), separation of the bone trabeculae (Tb.Sp), number of bone trabeculae (Tb.N), number of closed pores (Po-C.N), frequency of open porosity, frequency of total porosity (Po-tot), and connectivity (Conn).

After the analysis, the mean of the two measured values of each parameter, corresponding to the two sides of each mandible, was calculated for statistical analysis. A single examiner performed all measurements. The evaluations were performed on a single computer (Pentium (R), 3.40GHz and 2.25GB RAM), with Windows XP (Microsoft Corporation, Washington, United States) operating system, GeForce 6200 TurboCachel (NVIDIA, California, United States) video card, on a 20.1-inch (51 cm), SuperVGA (1024 × 768 pixels), 32-bit FlexScanS2000 SlimEdge (Eizo Nanao Corporation, Ishikawa, Japan) monitor.

Data were tabulated in specific Microsoft Excel spreadsheets. The sociodemographic variables (gender and age) were subjected to descriptive analysis by calculating means and standard deviations. Bone microstructure descriptors were compared using Student's *t* -test for paired samples. A 5% error probability was adopted for all analyses.

## Results

The sample consisted of 57 CT scans of people with SCD and 57 of people without bone changes, matched by age and sex. Thus, the mean age of the two groups was 37 years, with 28 men (49.1%) and 29 women (50.1%) in each group ( Table 1 ).

**Table 1:** Characterization of the sample groups regarding the age and sex of the patients

	Test group	Control group	*p* -Value
Mean age (min–max age)	37,1 (18–59)	37.2 (18–59)	0.57
18 to 30 years ( *n* [%])	18 (31.6%)		–
31 to 40 years ( *n* [%])	22 (38.6%)		–
41 to 50 years ( *n* [%])	7 (12.3%)		–
51 to 59 years ( *n* [%])	10 (17.5%)		–
Female ( *n* [%])	29 (50.1%)		–
Male ( *n* [%])	28 (49.1%)		–

Abbreviations: Min, minimum; max, maximum.

Of the total number of scans, 19 (33.3%) were obtained in cone-beam CT equipment (with isotropic voxel of 0.4 mm, 37 mA, and 120 kV) and the others (66.7%) were obtained in 64-channel multidetector CT equipment (0.5 × 0.3 mm, 150 mA and 120 kV).

To evaluate bone microarchitecture, the values of seven different mandibular bone pattern descriptors were compared. Three descriptors evaluated the configuration of trabecular bone (thickness of trabeculae, separation of bone trabeculae, and number of trabeculae), of which the last two showed significant difference between the groups evaluated. Bone porosity was estimated by three descriptors (number of closed pores, frequency of open porosity, and total porosity), and significant difference was observed between the groups for all three descriptors. The connection between the bone trabeculae was evaluated by connectivity. These results are shown in Table 2 .

**Table 2:** Descriptors of the mandibular bone pattern of the evaluated groups

	Test group—mean (SD)	Control group—mean (SD)	*p* -Value
Trabeculae thickness (Tb.Th)	3.45 (1.0)	3.22 (0.8)	0.20
Separation of trabeculae (Tb.Sp)	4.00 (1.2)	3.08 (1.2)	<0.0001 a
Number of trabeculae (Tb.N)	0.89 (0.8)	13.16 (10.2)	0.01 a
Number of closed pores (Po-C. N)	44.4 (58.3)	129.5 (184.7)	0.0026 a
Frequency of open porosity (Po-O)	57.5 (11.3)	47.5 (13.3)	<0.0001 a
Frequency of total porosity (Po-tot)	57.5 (11.3)	47.6 (13.2)	<0.0001 a
Connectivity (Conn)	633.3 (919.3)	880.9 (1291.1)	0.24

Abbreviation: SD, standard deviation.

a Significant difference.

## Discussion

Some studies using radiomorphometric parameters in two-dimensional images or in animal models have already been performed. 10 12 13 14 In our study, we observed a smaller number of bone trabeculae and a greater spacing between trabeculae in the group of people with SCD. These findings were observed in the study by Green et al 15 , with micro-CT in rats, the connectivity density showed a reduction in up to 80% when compared with the group without SCD. In this study, the authors found a reduction in the volume occupied by mineralized tissue in older animals with SCD. Thinner trabeculae with greater spacing between them and a smaller number of trabeculae were also found in animals with the disease.

Similarly, in the study by de Carvalho et al, 11 an increase in medullary spaces was observed in 67% of individuals with SCD on periapical radiographs, as well as in the study by Neves et al 16 who analyzed panoramic radiographs of individuals with SCD. In the study by Demirbas et al 10 panoramic radiographs were evaluated and in 67% of the patients with SCD, the quality of the mandibular bone tissue significantly deteriorated, trabecular bone density decreased, and the medullary spaces were enlarged and became locular. These authors also described a “ladder” trabecular pattern in 28% of the subjects analyzed, as well as thinning of the mandibular cortex in 22%.

When analyzing bone porosity, in our study a significant reduction in closed pores was observed, as well as an increase in open and closed porosity in people with SCD. Zlataric and Celebić 17 conducted a study using bone densitometry and panoramic radiographs, and found that individuals with low bone density values in the mandible had a much more porous cortical bone at the lower mandibular border.

In our study, no significant difference in bone connectivity was observed, although it was lower in people with SCD. In a study by Green et al 15 in which they analyzed micro-CT in rats, the connectivity density showed a reduction in up to 80% when compared with the group without SCD.

The imaging findings discussed here can be visualized on imaging scans as less dense and therefore more radiolucent tissue, with fewer radiopaque lines corresponding to the bone trabeculae. This can be explained in people with SCD by the higher medullary activity to contribute to increased hematopoiesis and compensate for the anemia that characterizes the disease.

This bone tissue configuration, although not unique to SCD, should be a warning sign for the underlying presence of diseases that may affect bone tissue metabolism.

Thus, this study proves the presence of mandibular bone changes in people with SCD, which may suggest the presence of this disease in people still undiagnosed and contribute to the screening of the disease.

## Conclusion

This study showed that the mandibular bone of people with SCD has lower bone density and more widely spaced trabeculae, which may contribute to the screening of people who have not been diagnosed with SCD or allow monitoring of the disease. This work is also important because knowing the mandibular bone microstructure of people with SCD impacts on dental therapeutical decisions, since we know that bone quality impacts directly in success in some procedures as surgeries and dental implants.
